# Feasibility study of streamlining radioembolization with yttrium-90 resin microspheres without lung shunt fraction measurement for colorectal liver metastasis < 7 cm

**DOI:** 10.1007/s11604-026-01984-w

**Published:** 2026-04-10

**Authors:** Myungsu Lee, Jin Chul Paeng, Jong Hyuk Lee, Minseok Suh, Sae-Won Han, Hyo-Cheol Kim

**Affiliations:** 1 Department of Radiology, Seoul National University Hospital, 101 Daehak-ro, Jongno-gu, Seoul, 03080 Republic of Korea; 2 Department of Nuclear Medicine, Seoul National University Hospital, 101 Daehak-ro, Jongno-gu, Seoul, 03080 Republic of Korea; 3 Department of Internal Medicine, Seoul National University Hospital, 101 Daehak-ro, Jongno-gu, Seoul, 03080 Republic of Korea; 4 Department of Radiology, Seoul National University College of Medicine, 103 Daehak-ro, Jongno-gu, Seoul, 03080 Republic of Korea; 5 Institute of Radiation Medicine, Seoul National University Medical Research Center, 103 Daehak-ro, Jongno-gu, Seoul, 03080 Republic of Korea

**Keywords:** Radioembolization, Colorectal liver metastasis, Streamlined treatment, Liver, Lung shunt fraction

## Abstract

**Purpose:**

To evaluate the feasibility of streamlining radioembolization using yttrium-90 resin microspheres without lung shunt fraction (LSF) assessment in patients with colorectal liver metastases (mCRC) < 7 cm.

**Materials and methods:**

This single-center retrospective study included 32 patients with mCRC who underwent radioembolization between June 2021 and April 2025. Eligibility criteria were: primary target tumor < 7 cm, the sum of the diameters of the two largest tumors ≤ 10 cm, treatment with resin microspheres, omission of LSF measurement, and at least one follow-up imaging study. Radiation activity was prescribed based on tumor location, liver function, and clinical setting, using both single-compartment and multi-compartment dosimetry with assumption of a LSF of 5% and tumor-to-normal (TN) ratio of 3. Post-treatment Y-90 PET/CT dosimetry was performed in 15 patients. Clinical outcomes, tumor response, and treatment-related toxicity were analyzed.

**Results:**

Median administered radiation activity was 0.50 GBq (IQR, 0.41–0.82). Median mean absorbed dose (mAD) to the perfused tissue was 136 Gy, and median tumor absorbed dose (TAD) was 347 Gy with assumption of TN ratio of 3. Post-treatment PET/CT analysis (n = 15) showed a median TAD of 432 Gy with a median TN ratio of 3.9, and lung dose derived from Y-90 PET/CT image ranged from 0.12 to 0.61 Gy. The best tumor responses were complete response in 12.5%, partial response in 59.4%, and stable disease in 28.1%. One- and two-year local tumor progression-free survival rates were 70.8% and 54.1%, respectively. No patient developed radiation pneumonitis.

**Conclusion:**

Streamlining radioembolization without LSF assessment appears feasible for patients with mCRC < 7 cm.

## Introduction

Radioembolization, also referred to as selective internal radiation therapy (SIRT), is considered one of the standard treatment options for patients with unresectable hepatocellular carcinoma (HCC) [1]. Radioembolization has been investigated as a treatment modality for liver dominant metastatic colorectal carcinoma (mCRC) [2, 3]. The recent EPOCH trial demonstrated that the addition of radioembolization to second-line chemotherapy improved hepatic progression-free survival in patients with mCRC who had progressed on first-line chemotherapy [4]. However, overall survival was not significantly prolonged in these clinical trials [3, 4].

A critical safety consideration in radioembolization is radiation exposure to the lungs resulting from hepatopulmonary shunting. To minimize the risk of radiation pneumonitis, established safety thresholds for pulmonary absorbed dose are commonly applied, including a maximum of approximately 30 Gy for a single treatment session and 50 Gy as a cumulative lifetime dose [5]. Conventionally, lung shunt fraction (LSF) is assessed using technetium-99 m–labeled macroaggregated albumin (MAA) scintigraphy during planning angiography to ensure that these safety margins are not exceeded.

The concept of streamlining radioembolization refers to performing radioembolization without formal LSF assessment in carefully selected patients, particularly those with small liver tumors and a low anticipated risk of significant hepatopulmonary shunting [6–8]. Between 2012 and 2021, the LSF in 32 patients with mCRC treated at the authors’ institution ranged from 1.4 to 16.1% (median, 3.1%; interquartile range [IQR], 2.0–5.7%), while in 24 patients with mCRC measuring less than 7 cm, the LSF ranged from 1.4% to 6.6% (median, 2.5%; IQR, 1.9–3.7%) (unpublished data). Importantly, these values corresponded to estimated pulmonary absorbed doses well below established safety limits.

On the basis of these institutional findings, since June 2021, most patients with mCRC smaller than 7 cm have undergone radioembolization with resin microspheres without preceding MAA scintigraphy and planning angiography. Thus, the purpose of this retrospective study was to evaluate the feasibility of streamlining radioembolization using resin microspheres in patients with mCRC measuring less than 7 cm.

## Materials and methods

### Patients

**Fig. 1:**
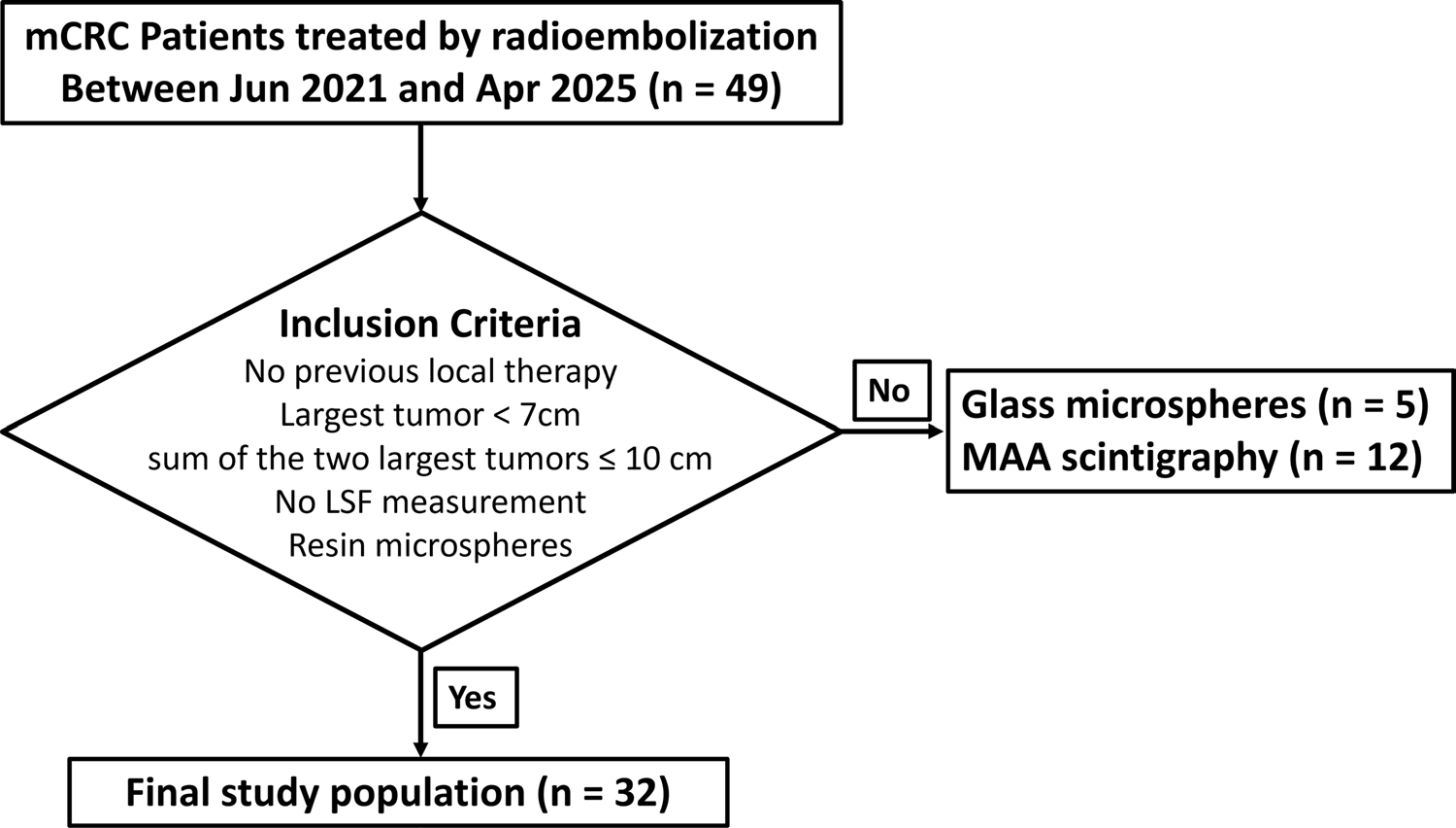
Study flow diagram showing patient exclusions. mCRC = metastatic colorectal cancer, MAA = macroaggregated albumin

This single-center retrospective study was approved by the institutional review board and received a waiver for informed consent. Between June 2021 and April 2025, a total of 49 patients with mCRC were treated with radioembolization. Inclusion criteria consist of no previous local treatment for the primary target tumor, the largest tumor size < 7 cm, the sum of the diameters of the two largest tumors ≤ 10 cm, no LSF measurement, radioembolization with resin microspheres (SIR-Spheres; SIRTEX, Woburn, MA, USA), and at least one follow-up imaging study (Fig. 1). Finally, 32 patients are included in this study. The baseline characteristics are summarized in Table 1. The size of the primary target tumor ranged from 1.3 to 5.5 cm (median, 2.7 cm; IQR, 2.1–3.4 cm), and 18 (56%) patients had single metastasis.

**Table 1:** Baseline demographics and treatment characteristics of the 32 patients

Characteristic	Value
*Sex*	
Male	19 (59)
Female	13 (41)
Age (years)^†^	64 (54–69)
*ECOG performance status*	
0	27 (84)
1	5 (16)
*Child–Pugh class*	
A5	29 (91)
A6	3 (9)
*History of smoking*	
Never smoking	28
1 ~ 10 pack year	2
More than 10 pack year	2
*Prior chemotherapy*	
None	1 (3)
1st line	15 (47)
2nd line	8 (25)
3rd line or more	8 (25)
*Previous angiogenesis inhibitor*	
yes	13 (41)
*Pretreatment laboratory values*^*†*^	
Aspartate transaminase (unit/L)	23 (20–30)
Alanine aminotransferase (unit/L)	19 (12–28)
Total bilirubin (mg/dL)	0.6 (0.3–0.8)
Albumin (g/dL)	4.1 (3.9–4.4)
Creatinine (mg/dL)	0.77 (0.70–0.89)
*Previous liver resection*	
Yes	17 (53)
*Concurrent metastasis*	
Lung	10
Peritoneal seeding	4
Lymph node	2
Bone	1
*Emphysema*	
Trace centrilobular emphysema	2
Mild centrilobular emphysema	1
Moderate centrilobular emphysema	1
Interstitial lung disease	0
*Tumor number*	
Single	18 (56)
Multiple	14 (44)
Tumor size (cm)^†^	2.7 (2.1–3.4)
< 2 cm	5
≥ 2 cm and < 3 cm	13
≥ 3 cm and < 4 cm	8
≥ 4 cm and < 5 cm	5
≥ 5 cm and < 7 cm	1
Total liver volume (mL)^†^	1068 (944–1341)
Treated liver volume (mL)^†^	244 (132–300)
Tumor volume (mL)^†^	10 (4–25)
*Treated vessel number*	
1	5 (16)
2	12 (38)
3	6 (19)
4	2 (6)
5	6 (19)
7	1 (3)
*Treated vessel level*	
Segmental artery	6 (19)
Segmental + Subsegmental artery	13 (41)
Subsegmental artery	13 (41)
Total radiation activity delivered (GBq)^†^	0.50 (0.41–0.82)
Mean absorbed dose to perfused tissue (Gy)^†^	136 (94–171)
Tumor absorbed dose (Gy)	347 (217–446)

Except where indicated, data are number of qualifying patients with percentages in parentheses

^†^Data represent median value, and numbers in parentheses are the interquartile range for continuous variables

### Radioembolization procedure

Radioembolization was performed by two interventional radiologists (H.C.K., and M.L., with 17 and 12 years of experience in interventional oncology, respectively). The segmental or subsegmental hepatic artery was catheterized with thin microcatheters with a 1.7-F tip (Progreat Lamda, Terumo, Tokyo, Japan). With resin microspheres, 3 days (n = 29) or 4 days (n = 3) pre-calibration dosing vial was used in all patients.

Identification of the tumor-feeding arteries, as well as the number of daughter vials and the prescribed radiation activity, was determined following hepatic angiography and cone-beam CT. The operator notified the nuclear medicine department about the radiation activity of each daughter vial, and then a technician prepared the daughter vials and provided the microspheres to the angiography suite within 20 min. The radiation activity of the smallest daughter vial was 0.1 GBq, and increased by 0.05 GBq. A mixture of half contrast and half 5% glucose solution was used as the injection fluid as well as the flushing fluid, and the legacy delivery system was used in all cases. Thus, the reflux of both injection and flushing fluid was monitored on fluoroscopy. Injection time commonly ranged from 3 to 5 min for one target vessel. When multiple vessels needed to be treated, a separate microcatheter was used for each vessel. Residual radiation activity was measured from all vials to calculate the total delivered radiation activity for each patient.

With assumption of a LSF of 5%, the upper limit of estimated lung dose was set as 15 Gy with the lung mass at 800 g for Asian men and 600 g for Asian women [9–11]. The required radiation activity was calculated by both single-compartment dosimetry and multi-compartment dosimetry. For single-compartment dosimetry, the mean absorbed dose (mAD) to the perfused tissue was targeted at 150–250 Gy. For multi-compartment dosimetry, assuming a tumor-to-normal (TN) ratio of 3, the tumor absorbed dose (TAD) was targeted at 300–600 Gy. When the tumor was located at the liver periphery, with Child–Pugh class A5, younger patient age, and no extrahepatic metastasis, the prescribed radiation activity was calculated to achieve both mAD of 250 Gy and TAD of 600 Gy. The final prescribed radiation activity was defined as the midpoint between the two calculated estimates. In contrast, when the tumor was centrally located in the liver, in older patients, or in a palliative setting (for example, extrahepatic metastasis was present), the prescribed radiation activity was determined based on achieving an mAD of 70–120 Gy and/or a TAD of 100–200 Gy.

### Dosimetry analysis

All radiation doses were calculated based on the actual delivered radiation activity rather than the prescribed activity. The mAD to the treated volume by single compartment dosimetry was calculated with assumption of a LSF of 5%. The TAD by multi-compartment dosimetry was calculated with assumption of a LSF of 5% and of a TN ratio of 3. Y-90 positron emission tomography (PET)/CT images were obtained in 15 patients the following morning. The TAD by multi-compartment dosimetry was calculated with the Y-90 PET/CT images with assumption of 5% LSF in these 15 patients. The commercially available software Simplicity^90^ (Mirada Medical, Oxford, Oxfordshire, UK) was used to perform post-treatment dosimetry.

Radiation doses to the lungs were calculated from post-treatment Y-90 PET/CT images in 15 patients, using a commercial software package (SurePlanTM LiverY90, MIM Software Inc., Cleveland, OH, USA). Volumes-of-interest (VOIs) for each lung were automatically generated on the CT images using an autocontouring tool, and subsequently applied to Y-90 PET images. When significant spill-over activity from the liver tumor was included, the VOIs were manually adjusted to minimize this effect. The radiation doses to the right and left lungs were calculated from the respective VOIs and averaged to yield the final lung dose.

### Clinical outcomes

All adverse events within the initial 90 days were assessed using electronic medical records and classified according to the National Cancer Institute Common Terminology Criteria for Adverse Events, version 5.0 [12]. Asymptomatic radiation pneumonitis was defined as the presence of new pulmonary abnormalities on chest CT scan (ground-glass opacities or consolidation with subpleural sparing, and fibrotic change on follow-up image), in the absence of respiratory symptoms. Symptomatic radiation pneumonitis was determined to be present when patients presented with exertional dyspnea, cough, and radiologic abnormality [13]. Radioembolization-induced liver disease (REILD) was defined as the development of jaundice and ascites without tumor progression or biliary tract obstruction [13].

Tumor response was assessed using Response Evaluation Criteria In Solid Tumors (RECIST) [14]. Continuous variables were compared using the t-test or Mann–Whitney U test. Local tumor progression-free survival (PFS) for the treated tumor, and overall survival were evaluated by Kaplan–Meier curves. A 2-sided *P* value of < 0.05 was considered to indicate a statistical significance. All statistical analyses were performed using SPSS version 25.0 software (SPSS, Inc., Chicago, IL, USA).

## Results

### Radioembolization

**Fig. 2:**
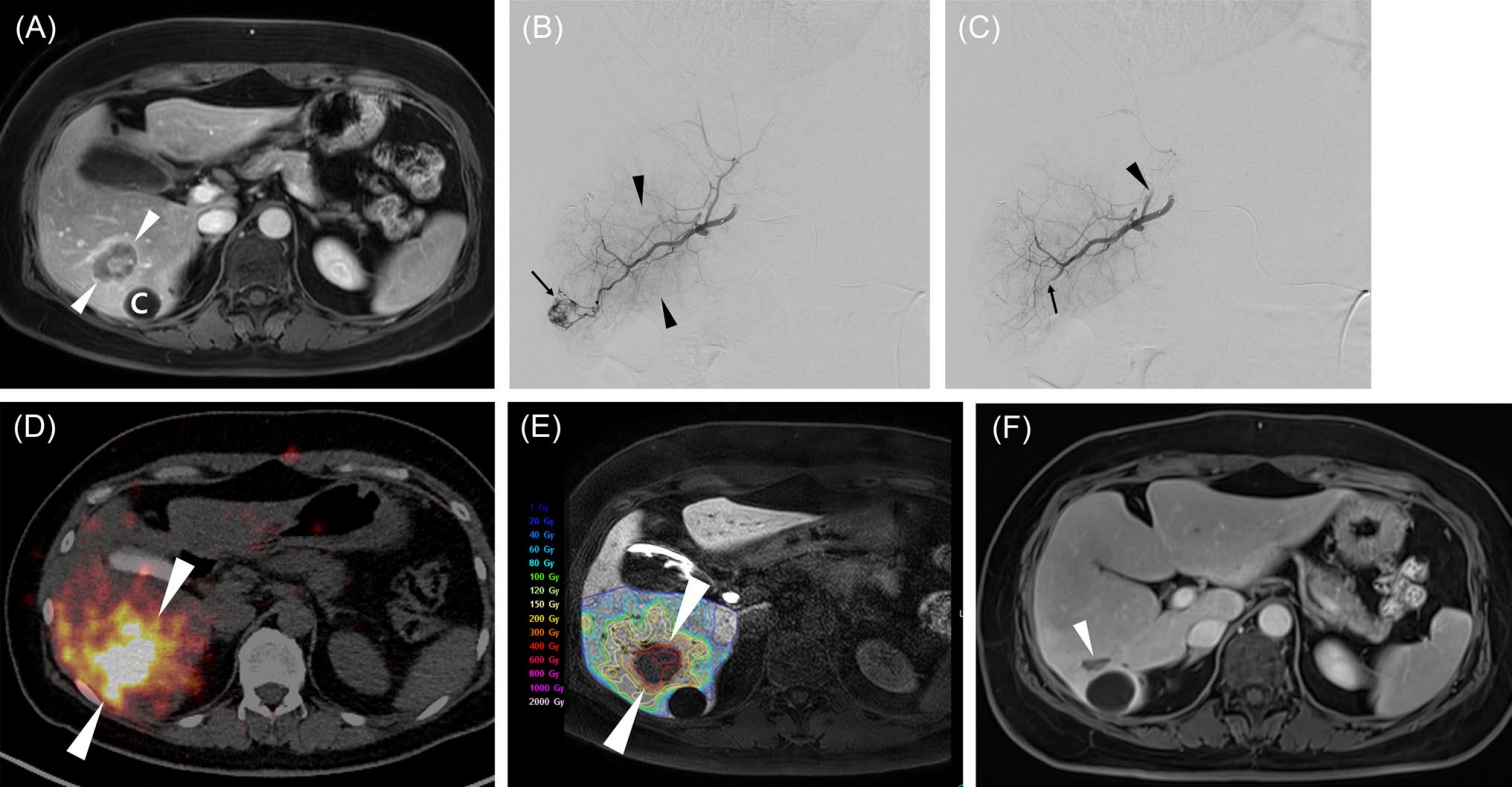
A 51-year-old woman with metastatic nodule from colon cancer. **A** Axial MR image of portal phase shows a 3.5 cm tumor in segment VI (arrowheads). Note simple hepatic cyst (C). **B** Selective angiogram of right posterior sectional artery shows tumor blush (arrowheads), and hemangioma (arrow). Segment VII artery and hemangioma was embolized with gelatin sponge particles. **C** Resin microspheres of 0.73 GBq were infused after embolization of segment VII artery (arrowhead) and hemangioma (arrow). **D** Y-90 PET/CT image obtained the next morning shows hot activity in the tumor of segment VI (arrowheads). **E** Dosimetric analysis image by Simplicity90 program shows hot activity in the tumor of segment VI (arrowheads). **F** Axial MR image of portal venous phase 6 months after radioembolization shows partial response of tumor in segment VI (arrowhead)

**Fig. 3:**
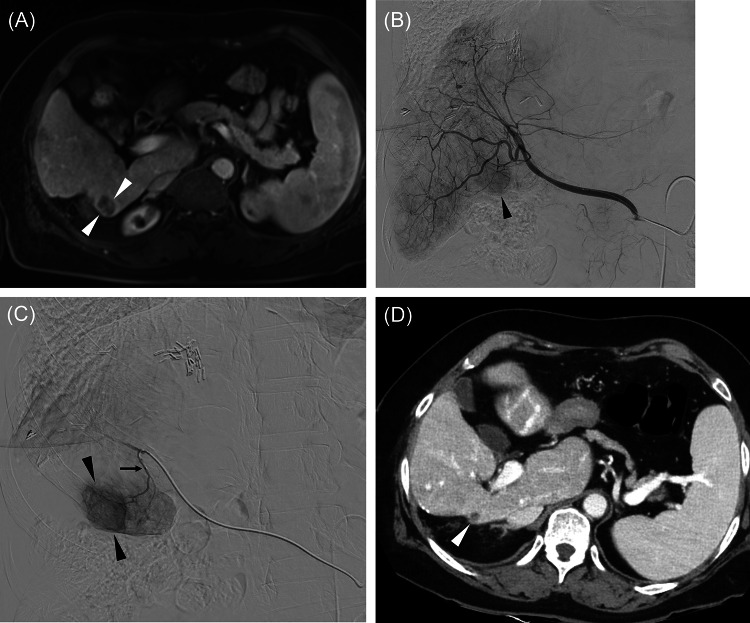
A 64-year-old woman with 2 cm sized single metastasis from colon cancer. She underwent low anterior resection and liver tumorectomy 11 years ago, followed by first-line chemotherapy. Six years ago, she experienced a recurrence with liver metastasis, for which tumorectomy was performed and second-line chemotherapy was administered. **A** Axial MR image of portal venous phase shows a 2 cm nodular tumor (arrowheads). **B** Right hepatic angiogram shows a tumor blush (arrowhead). **C** Selective angiogram of subsegmental branch shows small tumor blush (arrowheads). Resin microspheres of 0.4 GBq were infused at the subsegmental branch. Note a microcatheter tip (arrow). **D** CT scan 6 months after radioembolization shows partial response of the tumor (arrowhead)

Radioembolization was performed on 20 patients as an inpatient procedure, and 12 patients as an outpatient procedure. All 20 patients with inpatient procedure were discharged the next day. Total administered radiation activity ranged from 0.11 to 1.68 GBq (median, 0.50 GBq; IQR, 0.41–0.82 GBq). The number of total treated vessels ranged from 1 to 7 (median 2), and 4 or more vessels were treated in 9 (28%) patients. The level of treated hepatic artery was categorized into the segmental hepatic artery (n = 6) (Fig. 2), the segmental and subsegmental artery (n = 13), and subsegmental artery (n = 13) (Fig. 3) (Table 1).

According to the single compartment model with assumption of a LSF of 5%, the median mAD to the entire perfused tissue was 136 Gy (range 62–755 Gy, IQR, 94–171 Gy). According to the multi-compartment dosimetry with assumption of a LSF of 5% and of a TN ratio of 3, the median TAD was 347 Gy (range 160–1617 Gy, IQR, 217–446 Gy). Assuming an LSF of 5%, and the lung mass at 800 g for Asian men and 600 g for Asian women, the estimated lung dose ranged from 0.34 to 6.95 Gy (median, 1.94 Gy; IQR, 1.55–2.70 Gy).

According to the retrospective multi-compartment post-treatment dosimetry with PET/CT in 15 patients, the median TN ratio was 3.9 (range 1.7–9.4, IQR, 3.1–5.5), and the median TAD was 432 Gy (range 231–718 Gy, IQR, 273–597 Gy). In these 15 patients, the median TAD with assumption of a TN ratio of 3 was 331 Gy (range 182–758 Gy, IQR, 191–408 Gy). In these 15 patients, total delivered radiation activity ranged from 0.36 to 1.14 GBq (median 0.52 GBq; IQR, 0.42–0.91 GBq), and lung dose derived from Y-90 PET/CT image ranged from 0.12 to 0.61 Gy (median 0.28 Gy; IQR, 0.17–0.40 Gy).

### Clinical outcomes

**Fig. 4:**
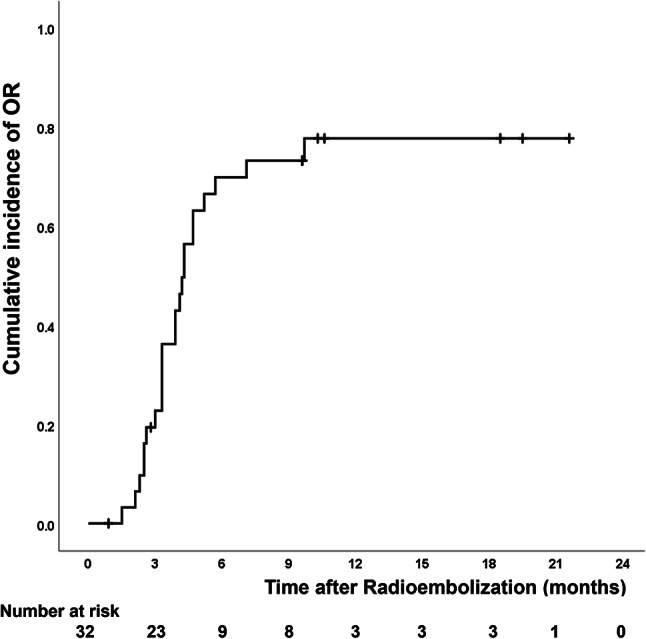
Cumulative incidence of radiologic objective response by RECIST

Median follow-up period of total study population was 16.2 months (95% CI, 11.2–21.2 months). The best response of treated tumor was complete response in 4 patients (12.5%), partial response in 19 patients (59.4%), and stable disease in 9 (28.1%). The median mAD values in the objective response (OR) group and the stable disease group were 136 Gy and 136 Gy, respectively, and the median TAD values were 352 Gy and 343 Gy, respectively. There was no statistically significant difference between the two groups (Table 2). The estimated probability of OR at 3 months and 6 months was 22.7% and 69.8%, respectively (Fig. 4).

**Table 2:** Best tumor response of the primary target tumor in 32 patients

All Patients (n = 32)	OR (n = 23)	SD (n = 9)	*P* value
mAD	136 (94–203)	136 (69–153)	0.289
TAD	352 (244–549)	343 (176–415)	0.211

Patents with PET/CT (n = 15)	OR (n = 11)	SD (n = 4)	*P* value
TAD	432 (290–577)	390 (252–574)	0.514

Data represent median value, and numbers in parentheses are the interquartile range for continuous variables

OR: objective response (complete response + partial response)

SD: stable disease

mAD: mean absorbed dose

TAD: tumor absorbed dose

**Fig. 5:**
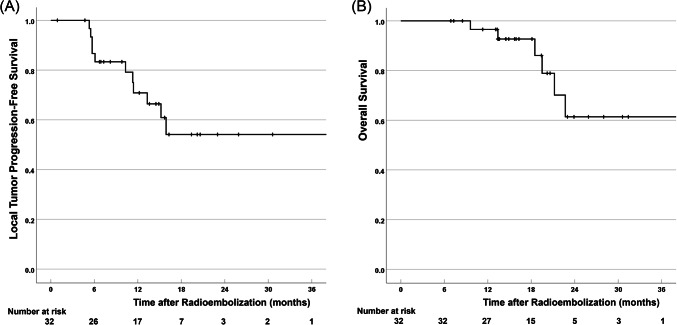
**A** Local tumor progression-free survival after radioembolization. **B** Overall survival after radioembolization

Local tumor PFS rates at 6 months, 1-year, and 2-year were 86.7%, 70.8%, and 54.1%, respectively (Fig. 5A). Median overall survival after radioembolization was not reached, and overall survival rates at 1-year, 2-year, and 3-year were 96.6%, 61.4%, and 61.4%, respectively (Fig. 5B).

### Safety

Clinical and laboratory toxicity was summarized in Table 3. All patients underwent at least one follow-up chest CT, and none demonstrated findings suggestive of radiation pneumonitis.

**Table 3:** Toxicity from radioembolization in 32 patients treated with radioembolization

Toxicity	Incidence	CTCAE grade			
		1	2	3	4
*Clinical toxicity*					
Pain	5	5			
Fever	0				
Nausea/Vomiting	2	2			
Fatigue	0				
Biliary duct dilation	0				
Ascites	0				
REILD	0				
Radiation pneumonitis	0				
*Biochemical toxicity*					
Decreased albumin	0				
Increased total bilirubin	2	1	1		
Increased AST	0				
Increased ALT	3	3			
Increased alkaline phosphatase	5	5			
Increased creatinine	1		1		
Decreased lymphocyte	6		5	1	

REILD = radioembolization-induced liver disease

AST = aspartate aminotransferase

ALT = alanine aminotransferase

## Discussion

This retrospective single-center study demonstrates that streamlining radioembolization with yttrium-90 resin microspheres—by omitting LSF assessment—can be performed safely and effectively in patients with mCRC smaller than 7 cm. The study adds real-world evidence to a growing body of literature suggesting that careful patient selection, tailored dosimetry, and contemporary delivery techniques may allow simplification of radioembolization workflows without compromising outcomes.

Traditionally, MAA scintigraphy is performed before radioembolization to estimate LSF and predict the risk of radiation pneumonitis [1, 15]. Although this step has long been considered standard, its necessity in selected patient populations has been questioned. In general, small tumors or mCRC tend to have a lower LSF compared with large tumors or HCC [16, 17]. Jakobs et al. reported a mean LSF of 4.9% in 104 patients with mCRC [18]. In authors institution’s historical cohort, the LSF in patients with mCRC was consistently low (median, 3.1%; interquartile range [IQR], 2.0–5.7%), and even lower among those with tumors < 7 cm. In this study, assuming an LSF of 5%, all patients had estimated lung doses below 7 Gy, which is far below the 25 Gy threshold (manufacturer’s recommendation) for pulmonary toxicity and also lower than authors’ institutional criterion of 15 Gy. In 15 patients with Y-90 PET/CT, the lung dose derived from Y-90 PET/CT image was less than 1 Gy in all patients. In addition, no findings suggestive of radiation pneumonitis were observed on follow-up chest CT in any patient. These observations support the concept that, in highly selected patients with mCRC < 7 cm, LSF assessment can be safely omitted.

The reason for selecting 7 cm as the cut-off value is based on our institutional experience, in which the highest LSF observed in tumors ≤ 7 cm was 6.6%. For example, assuming a tumor measuring 7 cm in diameter (approximately 180 mL in volume), a perfused volume of 500 mL, a T/N ratio of 3, a LSF of 5%, and a lung mass of 800 g, approximately 1.8 GBq should be administered to achieve a TAD of 300 Gy using the partition model. In this case, the estimated lung dose would not exceed 6 Gy which is far below the 25 Gy threshold (manufacturer’s recommendation). If two tumors measuring 6 cm and 5 cm are located separately in both lobes of the liver, both the total tumor volume and the perfused volume increase. As a result, the total radiation activity required also increases, and the estimated lung dose may consequently rise. However, if the size of the second-largest tumor is small, the total radiation activity required does not increase substantially. Therefore, for a single tumor, it is reasonable to set 7 cm as the upper limit. For multiple tumors, the authors think that it is necessary to limit the sum of the diameters of the two largest tumors to 10 cm.

Our cohort achieved high OR rates: complete or partial response in 71.9% of treated tumors and stable disease in the remainder. One- and two-year local tumor progression-free survival rates of 70.8% and 54.1% compare favorably with historical controls for second-line or salvage therapy. In addition, our findings suggest that when radioembolization is performed with high segmental or subsegmental selectivity and adequate dosimetry, meaningful tumor control and survival can be achieved even without formal LSF planning.

With the rapid increase in the number of radioembolization procedures, the nuclear medicine department has reached the limit of its capacity to accommodate MAA scans, resulting in prolonged waiting times and overall delays in the radioembolization treatment schedule. By omitting MAA scans in patients with small tumors, it becomes possible to perform radioembolization for patients with larger tumors—those who truly require MAA scanning—without treatment delays. In addition, at authors’ institution, superselective treatment via subsegmental branches is frequently performed. Although the absence of a planning angiography makes it impossible to know in advance the exact number of target vessels, resin microspheres offer the advantage that the nuclear medicine department can promptly prepare and deliver daughter vials as needed, making them well suited for streamlining radioembolization. In the present study, which included only patients with tumors < 7 cm, four or more vessels were treated in 28% of cases.

The body surface area model has been frequently applied in studies utilizing resin microspheres, and single compartment dosimetry was used in studies with glass microspheres [2–4, 19]. In addition, as most treatments were performed at the lobar level, the reported objective response rate was around 24–34% [2–4, 19]. Radiation segmentectomy with glass microspheres was reported for liver metastases, among which 31% of the patients had mCRC, and the partial response rate was 28% [20]. In a recent study of 36 patients with mCRC who underwent radiation segmentectomy, the median local tumor PFS was reported as 6.1 months, and a statistically significant improvement in local tumor PFS was observed when the mAD exceeded 400 Gy [21]. Doyle et al. reported that administration of an estimated TAD of 120 Gy predicted 55% OR with resin microspheres at 6 months [22]. In this study, the local tumor PFS at 6 months was 86.7% and the median local tumor PFS was not reached. In addition, the estimated probability of OR at 6 months was 69.8%. Given that the majority of patients in this study had small tumor size and either a single tumor or oligonodular metastases, direct comparisons with prior studies may not be appropriate. However, the favorable tumor response and excellent local PFS in this study may be attributed to the superselective treatment strategy applied in patients with a low tumor burden.

In this study, treatment was planned under the assumption of an LSF of 5% and a T/N ratio of 3. In radioembolization for mCRC < 7 cm, precise estimation of the LSF may not be critical because the variation in TAD is substantially smaller than the variation in LSF itself. For example, when LSF is calculated as 3% versus 9%, the LSF differs by a factor of three; however, if the same radiation activity is administered, the difference in TAD is within approximately 10%. However, the T/N ratio on PET/CT demonstrated a wide range (1.7–9.4) in this study. When single-compartment dosimetry is used, the T/N ratio is not incorporated into the calculation; therefore, a wide range of T/N ratios does not pose a significant issue. In contrast, with multicompartment dosimetry, the TAD varies considerably according to the T/N ratio, which may complicate determination of the appropriate therapeutic activity. Because the injections in this study were performed at the segmental or subsegmental arterial level, the treatment corresponds to radiation segmentectomy or subsegmentectomy, for which single-compartment dosimetry is generally applied. In our experience, however, the smaller the tumor, the greater the likelihood of overtreatment when single-compartment dosimetry alone is used. Therefore, we calculated both single- and multicompartment dosimetry and selected an intermediate value between the two. It is also feasible to adjust the T/N ratio based on tumor enhancement observed on angiography or cone-beam CT and to perform multicompartment dosimetry accordingly [23]; however, this approach requires further clinical experience and additional investigation.

This study has several limitations inherent to its retrospective single-center design. First, the sample size was small, which limits statistical power to detect dosimetric cut-off value predicting tumor response. Second, only 15 patients underwent post-treatment Y-90 PET/CT, which may introduce selection bias in dosimetric comparisons. Third, while our activity calculation assumed a conservative LSF of 5%, actual LSF values were not measured; thus, our estimated lung doses may underestimate true exposure in rare outlier cases. Fourth, heterogeneity in prior systemic therapy, tumor burden, and patient comorbidities may confound survival outcomes. Finally, long-term liver function and quality-of-life data were not systematically collected.

In conclusion, this study suggests that streamlining radioembolization with yttrium-90 resin microspheres without LSF assessment may be feasible in patients with mCRC smaller than 7 cm. Using a combination of single- and multi-compartment dosimetry, encouraging tumor response and local control were observed with acceptable toxicity. These findings warrant further validation in larger prospective studies.
